# Application of Dynamic Fragmentation Methods in Multimedia Databases: A Review

**DOI:** 10.3390/e22121352

**Published:** 2020-11-30

**Authors:** Felipe Castro-Medina, Lisbeth Rodríguez-Mazahua, Asdrúbal López-Chau, Jair Cervantes, Giner Alor-Hernández, Isaac Machorro-Cano

**Affiliations:** 1 Tecnológico Nacional de México/I. T. Orizaba, Division of Research and Postgraduate Studies, Av. Oriente 9 852. Col. Emiliano Zapata, C.P. 94320 Orizaba, Mexico; felipecastromedina123@gmail.com (F.C.-M.); galor@ito-depi.edu.mx (G.A.-H.); 2 Universidad Autónoma del Estado de México, Centro Universitario UAEM Zumpango, Camino viejo a Jilotzingo continuación Calle Rayón, Valle Hermoso, C.P. 55600 Zumpango, Estado de México, Mexico; alchau@uaemex.mx; 3 Universidad Autónoma del Estado de México, Centro Universitario UAEM Texcoco, Av. Jardín Zumpango, s/n, Fraccionamiento El Tejocote, C.P. 56259 Texcoco, Estado de México, Mexico; jcervantesc@uaemex.mx; 4 Universidad del Papaloapan, Circuito Central #200, colonia Parque Industrial, C.P. 68301 Tuxtepec, Oaxaca, Mexico; imachorro@unpa.edu.mx

**Keywords:** cost model, dynamic fragmentation, multimedia fragmentation, literature review, horizontal fragmentation, vertical fragmentation, hybrid fragmentation

## Abstract

Fragmentation is a design technique widely used in multimedia databases, because it produces substantial benefits in reducing response times, causing lower execution costs in each operation performed. Multimedia databases include data whose main characteristic is their large size, therefore, database administrators face a challenge of great importance, since they must contemplate the different qualities of non-trivial data. These databases over time undergo changes in their access patterns. Different fragmentation techniques presented in related studies show adequate workflows, however, some do not contemplate changes in access patterns. This paper aims to provide an in-depth review of the literature related to dynamic fragmentation of multimedia databases, to identify the main challenges, technologies employed, types of fragmentation used, and characteristics of the cost model. This review provides valuable information for database administrators by showing essential characteristics to perform proper fragmentation and to improve the performance of fragmentation schemes. The reduction of costs in fragmentation methods is one of the most desired main properties. To fulfill this objective, the works include cost models, covering different qualities. In this analysis, a set of characteristics used in the cost models of each work is presented to facilitate the creation of a new cost model including the most used qualities. In addition, different data sets or reference points used in the testing stage of each work analyzed are presented.

## 1. Introduction

Database fragmentation is a process for reducing irrelevant data accesses by grouping data frequently accessed together in dedicated segments [1]. The time consumed during the execution of queries in a parallel and distributed environment is highly affected by the form in which the tables comprising a database have been fragmented. The classical methods of fragmentation in a database distributed system helps to make the information retrieval faster and with smaller calculation efforts [2,3,4,5]. Three main fragmentation techniques have been defined for relational databases: horizontal fragmentation (HF), vertical fragmentation (VF), and hybrid or mixed fragmentation (MF). These techniques have been also extended for multimedia databases [1,6]. However, the problems of fragmenting multimedia databases require new approaches to solve them considering their characteristics (e.g., large size, content-based retrieval, changing access patterns). The fragmentation can be static or dynamic. In static partitioning, the database elements (attributes and/or tuples) are assigned to a fragment only once at creation time, then their locations are never changed. This approach has the following problems:

1. The database administrator (DBA) has to observe the system for a significant amount of time before the partitioning operation can take place until the probabilities of queries accessing certain database elements and their frequencies are collected, this is called an analysis stage.
2. Even then, after the partitioning process is completed, nothing guarantees that the real trends in queries and data have been discovered. Thus the partitioning scheme may not be good. In this case, the database users may experience a very long query response time.
3. In some dynamic (e.g., multimedia) applications, queries tend to change over time and a partitioning scheme is implemented to optimize the response time for one particular set of queries. Thus, if the queries or their relative frequencies change, the fragmentation result may no longer be adequate.
4. In static partitioning methods, refragmentation is a heavy task and only can be performed manually when the system is idle

In contrast, in dynamic vertical partitioning, attributes are being relocated if it is detected that the current vertical partitioning scheme has become inadequate due to query information changes. [7].

Multimedia data are very important in many application areas such as medicine [8,9,10], cartography [11,12,13], meteorology [14,15,16], security [17,18,19], among others. Automatic extraction, classification, and manipulation of multimedia content are critical to efficient multimedia data management. Content-based media data retrieval methods improve the accuracy of database searches. These methods are necessary when the textual annotations are missing or incomplete. In addition, content-based methods potentially improve retrieval accuracy even when textual annotations are present, by giving additional insight to collections of multimedia data [20,21].

This work provides an in-depth review of works related to database fragmentation, with special emphasis on methods that focus on multimedia data, that perform dynamic fragmentation, and that contemplate CBIR (Content-Based Image Retrieval). Furthermore, different details of each work analyzed are shown, such as whether it is complete for its full implementation, whether the characteristics of the method makes it easy to implement, the main qualities of the cost model used, the technologies used in the implementation stage and the benchmarks used in the evaluation.

This article stands out for being the only one to contemplate the specific characteristics mentioned above, deeply reviewing the literature of dynamic fragmentation in multimedia databases to identify areas of opportunity aimed at clear recommendations for the creation of new methods.

This paper is structured as follows. Section 2 describes the research methodology used in this study. Section 3 presents the classification and analysis of the works obtained in the search stage. Section 4 gives a classification of fragmentation papers considering six interesting criteria: (1) year of publication; (2) editorial; (3) type of fragmentation, (4) used costs, (5) Database Management System, and (6) used benchmarks. Section 5 provides a description of the set of works by category. A discussion is included in Section 6. Finally, Section 7 presents conclusions and future directions.

## 2. Research Methodology

**Figure 1:**
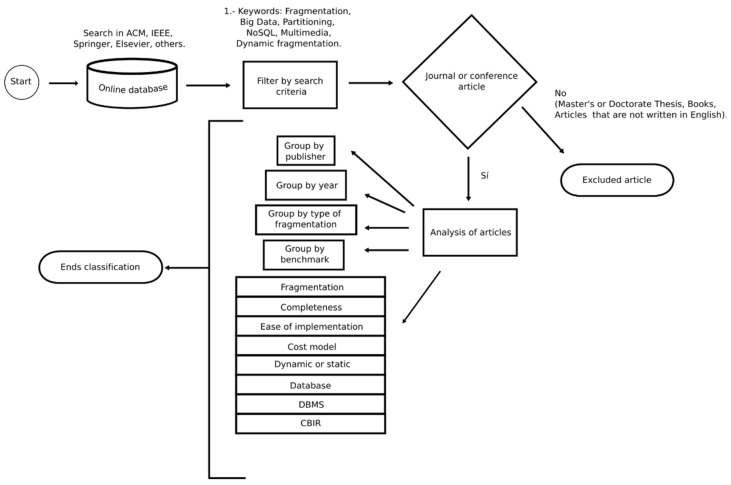
Research methodology flow diagram.

Dynamic fragmentation is a highly addressed topic in the literature of works related to fragmentation focused on improving the performance of distributed databases. To give an approach that contributes to the main characteristics of this work, it is necessary to know the related works. In Figure 1, all the stages of the proposed methodology are shown. The search for works was carried out in the main digital libraries of scientific publishers, ACM Digital Library, IEEE Xplore Digital Library, SpringerLink, and Science Direct (Elsevier). The works found that are not published by said editorials are categorized in “Other”. The search consists of finding all the papers that contain the following keywords: Fragmentation, Big Data, Partitioning, NoSQL (Not Only SQL), Multimedia, Dynamic Fragmentation. Once all the works were obtained, a filter was applied and all the works that are master’s or doctoral thesis, as well as books and articles that are not written in English, were discarded. The resulting articles were classified by the publisher, by year, by type of fragmentation, by benchmark and the most used costs are described. Each article is analyzed in detail indicating the type of fragmentation, if it has all the details to reproduce the method, if the elements used are easy to implement, if it has a cost model, also it is detailed if the fragmentation is static or dynamic, which type of database was handled, which Database Management System (DBMS) was handled and, finally, if the method contemplates CBIR.

## 3. Classification Method

80 articles were obtained in the search stage and they were analyzed according to the eight criteria presented in Figure 1. Table 1 shows the description of the methods exposing the type of fragmentation used, if it presents a cost model, if it is dynamic, if it considers CBIR, and, finally, the cost equation used to obtain the fragmentation scheme.

## 4. Classification of Research Papers

After having obtained the works of the search methodology, an analysis was carried out using graphs and tables that show a clear guide for future research.

**Figure 2:**
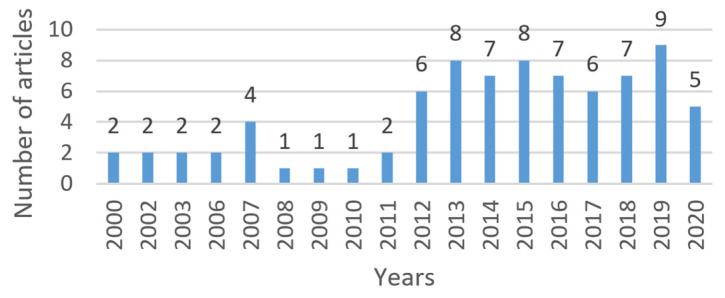
Number of articles by year of publication.

**Figure 3:**
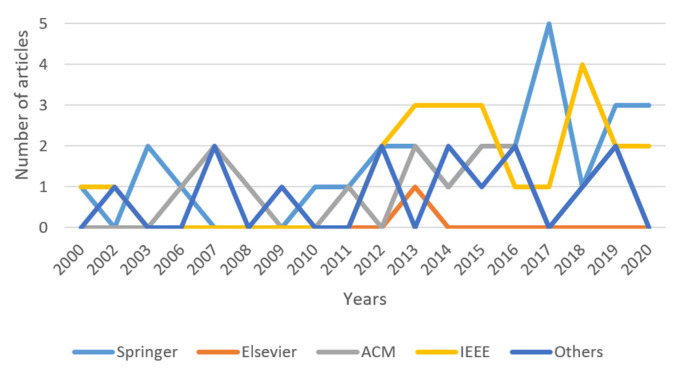
Number of articles by year of publication for each publisher.

**Figure 4:**
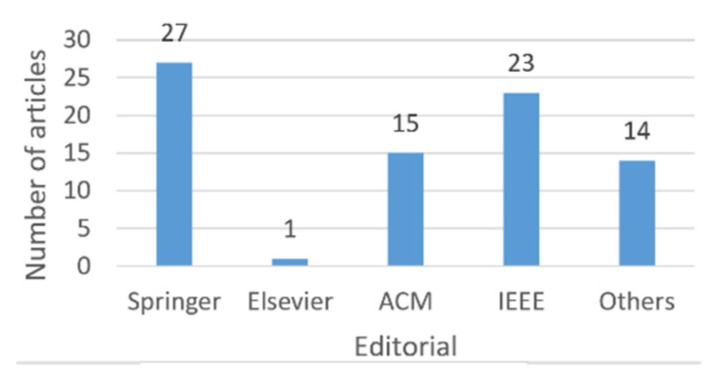
Number of articles by editorial.

Figure 2 shows a bar graph with the distribution of the articles analyzed according to the year of publication. It is observed that in 2019 the largest number of articles is concentrated. Of the 9 articles, 3 are from Springer ([33,34,66]), the publisher with the highest number of articles, as it can be observed in Figure 3 and Figure 4; 2 belong to ACM ([76,78]); 2 to IEEE ([35,96]), and 2 pertain to the category “Others” ([95,97]).

Figure 4 shows a bar graph that relates the number of articles to the publisher; most of the articles are concentrated in the Springer publishing house. Springer’s articles primarily addressed vertical ([22,24,25,26,32,33,34,36]) and horizontal ([1,43,46,47,51,55,58,59,63,66]) fragmentation, 18 of 26 articles. Only one article considers both fragmentation and CBIR ([1]).

**Figure 5:**
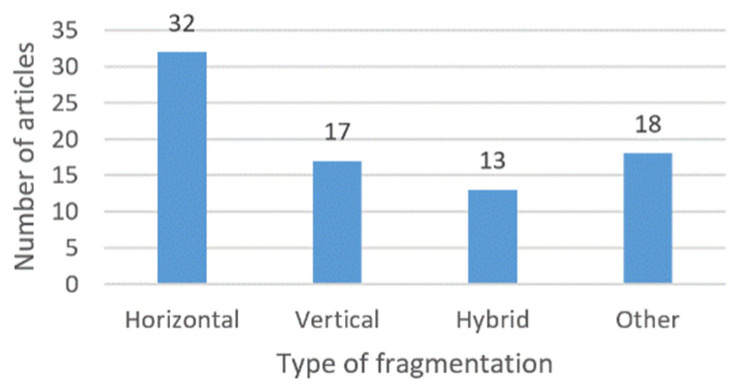
Number of articles by type of fragmentation.

Figure 5 depicts a bar graph showing the number of articles by type of fragmentation. The most used fragmentation in the analyzed works is the horizontal one. The “Other” category shows the number of articles that use a type of fragmentation that is not any of the other three, such as the fragmentation of documents in [82,83,84,85,86,87], fragmentation of videos in [88], grid fragmentation in [95], among others. Of the papers that addressed horizontal fragmentation, 4 also included CBIR ([1,40,41,49]).

Table 2 shows the costs that were found throughout the analysis and the articles that use them. Different articles contemplate more than one type of cost, for this reason, the total number of articles in the second column is not the total number of articles that were analyzed. The cost of transportation, access, storage, and execution are the most used, with a total of 63 mentions in the articles.

**Figure 6:**
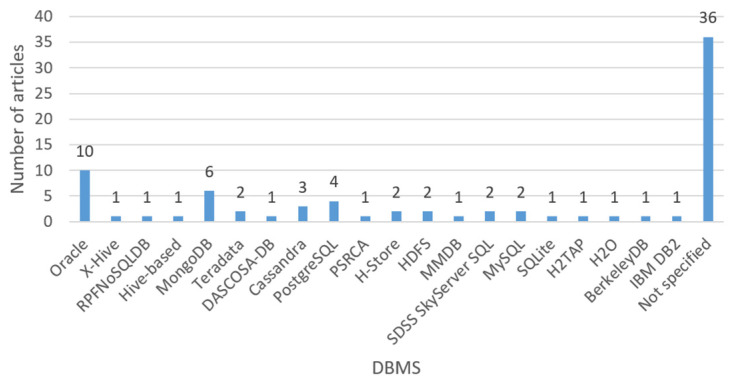
Number of articles by DBMS.

Figure 6 shows the number of articles per DBMS. Most of the works do not report or do not use a DBMS to carry out their research. It is observed that the most used systems are Oracle, MongoDB, PostgreSQL, and Cassandra.

Table 3 shows the most used benchmarks, which, likewise, the right column does not reflect the number of total articles, since many articles use more than one benchmark. The TPC-H benchmark is the most widely used.

**Figure 7:**
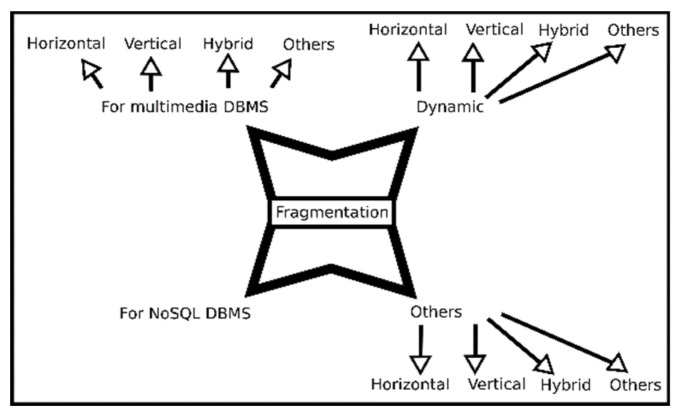
Classification of the articles obtained in the search stage.

One way to understand the different approaches of the works obtained is to classify them using the categories shown in Figure 7.

Four categories are proposed to present the works obtained. The first category contains the methods that focus on multimedia databases; the second category covers all the approaches that perform a dynamic fragmentation and that do not focus on multimedia data; the third category contains all the works that consider NoSQL databases, that do not perform dynamic fragmentation and that do not take into account multimedia elements; the last category shows the works that are not included in any of the previous categories. Each category is subdivided by type of fragmentation, i.e., horizontal, vertical, and hybrid. The third category is not subdivided due to the nature of NoSQL databases.

NoSQL technology has high availability and high scalability, which provides new methods for the storage and management of unstructured data. NoSQL technology abandons the relational model of paradigm constraint, can store data with different types, and has high scalability characteristics [61]. These features make NoSQL databases attractive for storing and managing multimedia data.

The two categories shown at the top of the image are the characteristics on which this work mainly focuses. The set of works for each category is presented in the next section.

## 5. Description of the Set of Works by Category

This section details each article in each of the categories, mainly presenting implementation characteristics, the type of fragmentation, and the results obtained.

### 5.1. Fragmentation of Multimedia Databases

Currently, multimedia applications are highly available, such as audio/video on demand, digital libraries, electronic catalogs, among others. The rapid development of multimedia applications has created a huge volume of multimedia data and it is exponentially incremented from time to time. A multimedia database is crucial in these applications to provide efficient data retrieval [55]. In this section, the works that address fragmentation focused on multimedia databases are grouped.

#### 5.1.1. Horizontal Fragmentation of Multimedia Databases

Several authors focus on horizontal fragmentation for multimedia databases, however, all propose different ways to carry out this task. In [39], Ma et al. analyzed fragmentation and allocation in the context of databases with complex values. The main contribution of the authors was a heuristic approach to fragmentation and allocation. The implementation was carried out using a database scheme which was populated by benchmark 007, subsequently, four sites and 30 queries were considered, of which 20% of them were frequently used for the most important transactions. The result of the experiment validated the proposed approach since the cost of transportation was minimized and, in this way, the performance of the database schemes was improved.

In [40,41], the primary horizontal fragmentation of textually annotated multimedia data was addressed. In these works, the problem of identifying the semantic implications between textually based multimedia predicates was observed and it was proposed to integrate knowledge bases as a framework to evaluate the affinity semantics between values of predicates and operators. The implementation consisted of varying the number of predicates and the number of concepts to obtain the execution time using the semantic base of predicates. Operator implications are identified using a specific knowledge-based operator, developed in [41]. In addition, a prototype was presented to test the approach used, which demonstrated that the proposed method has polynomial complexity.

In [49], Fasolin et al. proposed an efficient approach to running conjunctive queries on complex big data along with conventional data. A horizontal fragmentation was performed according to the criteria frequently used in the query predicates. This strategy was applied in CoPhIR, a collection of more than 106 million images along with its related conventional data. The experimental results showed a considerable increase in performance with the proposed approach for queries with conventional and similarity-based predicates, compared to the use of a single metric index for the entire content of the database.

Rodríguez-Mazahua et al. [55] developed a horizontal fragmentation method for multimedia databases which is based on an agglomerative hierarchical clustering algorithm. The main advantage of this method is that it does not use affinity to create a horizontal fragmentation scheme. In this work, the multimedia horizontal fragmentation algorithm was described using an example of equipment management in a machinery sales company and the cost model was presented in detail. The algorithm evaluation was carried out and it was shown that it exceeds the performance to create the fragmentation scheme in most cases.

Hakka culture is an important part of the culture of southern China. NoSQL technology has high availability and high scalability, this provides new methods for data storage and management of Hakka culture. In [61], Wu, Chen & Jiang managed unstructured data of the Hakka culture using MongoDB. Data from the Hakka culture of western Fujian were taken as an example and the prototype data management system was built. The authors concluded by mentioning that a new method was presented to improve the flexibility and efficiency of managing unstructured and heterogeneous data from multiple sources.

#### 5.1.2. Vertical Fragmentation for Multimedia Databases

Fung, Leung & Li [24] described the development of a video eLearning database system. The video eLearning database provides a framework for temporal modeling to describe video eLearning data and supports the distribution of data by applying vertical class fragmentation techniques. Vertical class fragmentation techniques were applied over 4DIS (Four-Dimensional Information System) as a measure for efficient query execution. The dynamic acquisition of eLearning multimedia video on the internet was presented and a detailed cost model was developed for the execution of queries through vertical class fragmentation. The effectiveness of this approach was demonstrated in the context of the video eLearning database system using the 4DIS modeling framework.

The authors in [26] presented a vertical fragmentation algorithm for distributed multimedia databases (MAVP, Adaptable Multimedia Vertical Partitioning) that takes into account the size of the multimedia objects to generate an optimal vertical fragmentation scheme. MAVP minimizes the number of irrelevant data accesses and the transportation cost of queries in distributed multimedia databases to achieve efficient retrieval of multimedia objects. This paper presented the development of a cost model that considers the cost of general query processing in a distributed multimedia environment. The experimental evaluation showed that it outperformed AVP [98], an algorithm with the same approach.

The vast majority of vertical fragmentation algorithms are static, that is, they optimize vertical fragmentation schemes using a workload, but if it undergoes changes, the schema will also suffer, resulting in long query response times. In [32], Rodríguez-Mazahua et al. proposed a set of active rules to perform dynamic vertical fragmentation on multimedia databases. Active rules are implemented in DYMOND (DYnamic Multimedia ONline Distribution), which is a system based on active rules for dynamic vertical fragmentation of multimedia databases. In this work, a case study of a machinery sales company was presented, which has a multimedia database, this database contains a table called equipment and contains 100 tuples with alphanumeric and multimedia attributes. Vertical fragmentation was performed applying the active rules on this database and a shorter response time was obtained when the first fragmentation was performed and also when the database was refragmented.

#### 5.1.3. Hybrid Fragmentation for Multimedia Databases

In [67], Chbeir and Laurent presented a formal approach to identify implications of predicates and queries to efficiently fragment multimedia data. The authors in this work focused on the fragmentation of databases with the objective of reducing access to irrelevant data by grouping frequently used data. The proposed approach uses combined fragmentation of multimedia data based on query comparison and query equivalence. Multimedia functional dependencies take into account specific characteristics of multimedia data and are axiomatized in the same way as standard functional dependencies. An example of a multimedia database is presented as an implementation that considers a table called “Albums” and contains the attributes name, birth, place, genre, image, song, and clip. Fragmentation is applied to that table.

Due to the increase in multimedia applications, the use of fragmentation techniques to reduce the number of pages required to answer a query is very useful. In [73], Rodríguez-Mazahua et al. presented a hybrid fragmentation method for multimedia databases that takes into account the size of the attributes and the selectivity of the predicates to create hybrid fragmentation schemes. The proposed algorithm searches for the most suitable vertical partition scheme to later obtain the hybrid scheme taking into account the cost model described in the same work. To carry out the experiments, the same scenario was used as in [32]. As a result, the hybrid partition method for multimedia databases was obtained, which reduces access to irrelevant data, taking into account the size and selectivity of each attribute, and also presented a cost model for distributed multimedia databases.

In [74], the authors created an index partition algorithm that addressed the specific properties of a distributed system: load balancing between nodes, redundancy in node failure, and efficient use of nodes under concurrent queries. The experiments focused on measuring the effectiveness of partition size balance and recovery quality. The results show that B-KSVD (Balanced K-means-based Single Value Decomposition) better balances partition sizes, compared to k-means and KSVD. In conclusion, it was mentioned that the requirements to create complete representations with redundant document indexing were formalized, where partitions contain overlapping data subsets.

Vogt, Stiemer & Schuldt [77] presented Polypheny-DB’s vision of a distributed polystore system that seamlessly combines replication and partitioning with local polystore and can dynamically adapt all parts of the system when workload changes. The basic components for both parts of the system were presented and the open challenges towards the implementation of the Polypheny-DB vision were shown.

Different domains take care of managing massive data volumes and thousands of OLTP (OnLine Transaction Processing) transactions per second. Traditional relational databases cannot cope with these requirements. NewSQL is a new generation of databases that provides high scalability, availability, and support of ACID properties (Atomicity, Consistency, Isolation, Durability). Schreiner et al. [78] proposed a hybrid fragmentation approach for NewSQL databases that allows the user to define vertical and horizontal data partitions. The experimental evaluation compared the hybrid version of VoltDB with standard VoltDB. The results highlight that the strategy shown increased the number of transactions in a single site from 37% to 76%, maintaining the same response time.

#### 5.1.4. Other Types of Fragmentation for Multimedia Databases

Different papers address other types of fragmentation for multimedia databases. In [82], the authors carry out hierarchical multimedia fragmentation for XML (Extensible Markup Language) documents. The results showed that the textual information of the elements must be different and returning multimedia fragments based on the hierarchical relationships between the common elements and the multimedia elements allows obtaining good results.

Torjmen-Khemakhem, Pinel-Sauvagnat & Boughanem [84] studied the impact, in terms of effectiveness, of the position of the text in relation to the sought multimedia objects. The authors’ general approach was based on two steps: first, it retrieves XML elements containing multimedia objects, and then, explores the surrounding information to retrieve relevant multimedia fragments. Different experiments were carried out in the context of the INEX framework (Initiative for the Evaluation of XML Retrieval), which contains more than 660,000 XML documents. The results showed that the structural evidence is of great interest to adjust the importance of the textual context for multimedia recovery.

In [86], Santos & Masala proposed a novel approach that combines a pattern fragmentation technique with a NoSQL database to organize and manage fragments. The experiments were carried out at different cloud service providers and three types of files were used, docx, jpg, and pdf, all with 100 kb in size. The result showed that fragmentation with random patterns is faster than other approaches.

Mourão & Magalhães [87] described how sparse hashes can help find an index where partitions are based on the feature vector distribution in the original space and create better distribution options for high dimension feature vectors. Different focus tests were performed on a commercial cloud service. It was tested on a billion vector dataset showing that this approach had low fragmentation overhead, achieved balanced document and query distribution, handled concurrent queries effectively, and had little degradation when nodes failed.

In [88], Mettes et al. proposed to fragment videos by recognizing events within them, and through this technique, they improved calls to sub-events. The experiments were carried out on a data set called THUMOS’14, which contained 1010 videos. The experimental evaluation showed the effectiveness of the coding of event detection as well as the natural complementation of the global aggregation of semantic concepts.

Lu et al. [94] designed a new index structure called Dynamic Partition Forest (DPF) to hierarchically divide high collision areas with dynamic hashing, to cause self-adaptation of various data distributions. The results of the experiment showed that DPF increases accuracy by 3% to 5% within the same period compared to DPF without the multi-step search. Experimental comparisons with two other leading-edge methods on three popular data sets show that DPF is 3.2 to 9 times faster to achieve the same precision with a decrease in index space from 17% to 78%.

The authors in [95] analyzed DICOM (Digital Imaging and Communications in Medicine) to find the optimal hybrid data configuration. NSGA-G (Non-dominated Sorting Genetic Algorithm—Grid) based on grid fragmentation was proposed to improve queries to hybrid DICOM data. Experiments on DICOM files on hybrid storage prove that NSGA-G provides the best processing time with interesting results.

The authors in [97] presented a cultural Big Data repository as an efficient way to store and retrieve cultural Big Data. The proposed repository is highly scalable and provides high performance integrated methods for Big Data analysis of cultural heritage. The experimental results show that the proposed repository exceeds in terms of space, as well as the storage and recovery time of cultural Big Data.

### 5.2. Dynamic Fragmentation

Dynamic fragmentation, as detailed in Section 1, improves the performance of databases, solving different problems of a static approach. This section shows all the works that focus on performing dynamic fragmentation that is not oriented to multimedia databases.

#### 5.2.1. Dynamic Horizontal Fragmentation

Vazquez [2] presented a method of Dynamic Virtual Fragmentation. The proposed method was tested and implemented on a parallel database server running on a supercomputer with shared-nothing architecture. By implementing the proposed method, performance improvement in information retrieval was achieved in queries that did not contain any attribute used by the horizontal fragmentation of the table in the selection criteria.

In [38], the authors addressed the problem of dynamic data reallocation in a distributed, fragmented database with changing access patterns. The algorithm was implemented on the database of the Federal Electricity Commission, which had changeable access patterns. As a result, it was observed an excellent performance of the database under the proposed approach.

A decentralized approach to fragmentation and dynamic table allocation was proposed in [43] for distributed database systems based on observation of site access patterns to tables. This approach, called DYFRAM, was evaluated in three stages. The first phase was to examine the results from running a simulator on four workloads involving two sites. In the second part of the evaluation, the same simulator was used with two dynamic workloads involving more sites. The third part of the evaluation consisted of implementing the experiments on a distributed database system. Simulation results proved that, for typical workloads, DYFRAM significantly reduces communication costs.

Abdalla & Amer [45] proposed a synchronized model of horizontal fragmentation, replication, and allocation in the context of relational databases. The experiments were carried out using a table, which contained information about different employees of a company. Four sites were considered on a distributed system, the costs between sites, and the restrictions of each one. This work significantly improved the performance of distributed database systems by reducing remote access and high costs of data transfer between sites.

In [46] and [51], the authors designed DynPart and DynPartGroup, two dynamic fragmentation algorithms for continuously growing databases. The solution was validated through experimentation on real data. The dataset was taken from the Sloan Digital Sky Survey catalog, Data Release 8 (DR8). The results show that in the case of data sets in which there is a high correlation between the new data elements, the DynPartGroup algorithm maintains very good behavior.

In [47], Bellatreche et al. proposed and experimentally evaluated an incremental approach to select data storage fragmentation schemes using genetic algorithms. The proposed approach was evaluated using the APB1 benchmark and a data warehouse with a star schema, which was populated with more than 24 million tuples and 4-dimensional tables. The tests were carried out on a small scale and on a large scale, which showed that of the three algorithms compared, the one proposed by the authors, called ISGA (Incremental Approach Based on Genetic Algorithms), outperformed the other two.

Derrar, Nacer & Boussaid [48] developed an approach based on the statistical use of data access for dynamic data fragmentation in data warehouses. Experimental studies were performed using Oracle 10 G with the APB1 benchmark to verify the adaptability of the proposed approach. Several tests were performed considering the evaluation criteria as a threshold for the response time of an OLAP query. The results obtained were encouraging both in terms of memory space and query execution time.

Herrmann, Voigt & Lehner in [52] solved the problem of online fragmentation for irregularly structured data and presented Cinderella, an autonomous online algorithm for horizontal fragmentation of irregularly structured entities in universal tables. The evaluation was carried out with the TPC-H and DBpedia benchmarks. Cinderella was implemented in PostgreSQL.

Kumar & Gupta [53] designed an algorithm called TTVDCA (Threshold, Time, Volume, and Distance Constraint Algorithm) for dynamic fragment allocation in non-replicated distributed database systems. Calculations on the hypothetical database supported that the algorithm is better than other algorithms previously developed in this category and showed an improvement in overall system performance.

The innovation presented by Baron & Iacob in [54] consisted in the possibility of integrating the three specific fundamental concepts of distributed databases: fragmentation, replication, and fragment allocation in an unbalanced, fully decentralized, and fully automated dynamic system. The authors mentioned that they not only innovated but also improved performance with the proposed approach which is configurable and easy to manage.

Fetai, Murezzan & Schuldt [56] presented Cumulus, an adaptive data fragmentation approach that can identify characteristic access patterns of transaction mixes, determine data partitions based on these patterns, and dynamically re-fragment data if access patterns change. The approach evaluation was performed with the TPC-C benchmark and it was shown that Cumulus significantly increased overall system performance in an OLTP configuration compared to static data partitioning approaches.

Abdel et al. [58] developed an improved dynamic system of distributed databases on a cloud environment, which allows them to dynamically make fragmentation, allocation, and replication decisions at runtime. Experiments were performed on a table containing data on bank user accounts. Three sites were used to apply the fragment distribution. An efficient approach to fragmentation, allocation, and replication based on access history was presented. The objective of the presented technique is to maximize local access.

Serafini et al. [60] presented a new online fragmentation approach, called Clay, that supported both tree-based schemas and more complex general schemas with arbitrary foreign key relationships. To evaluate the proposed approach, Clay was integrated into a distributed main memory DBMS and it was shown that it can generate partition schemes that allow the system to achieve up to 15 times better performance and 99% lower latency than existing approaches.

Zar Lwin & Naing [65] proposed an approach for the dynamic allocation of non-redundant fragments in a distributed database system. The implementation was carried out on four fully connected cloud sites, in which 10,000 queries were executed. The proposed approach was used to fragment the database and migrate each fragment to the best site. The result of the experiment showed that the performance of the four sites after the migration is slightly better than the performance of the four sites before migration.

Olma et al. [66] presented an online partitioning and indexing scheme, along with a partitioning and indexing tuner designed for in situ query engines. An on-site query engine called Slalom was created to show the impact of the proposed design. As a result of its lightweight nature, Slalom achieves efficient query processing on raw data with minimal memory consumption. The authors showed at the implementation stage that Slalom outperforms the latest generation in situ engines using microbenchmarks and actual workloads.

#### 5.2.2. Dynamic Vertical Fragmentation

Rodriguez et al. [7] discussed the improvement in DYVEP (DYnamic VErtical Partitioning), which was developed as an active system with dynamic partitioning capacity. The implementation was performed on the PostgreSQL database and the TPCH benchmark was used. The results showed the improvement in performance that DYVEP has when applied and all the advantages of the proposed approach were considered.

Pérez et al. [22] proposed an extension of the DFAR (Dynamic Fragmentation, Allocation, and reallocation) mathematical optimization model, which unifies the fragmentation, allocation, and dynamic migration of data in distributed database systems. The extension consisted of adding a constraint that models the storage capacity of network sites. Initial experiments revealed that when site capacity is used almost to its limit, attribute deadlocks can occur, preventing the threshold acceptance algorithm from converging.

Ref. [28,30] show the SMOPD (Self-Managing Online Partitioner for Databases) and SMOPD-C (Self-Managing Online Partitioner for Distributed Databases on Cluster Computers) algorithms that can autonomously partition a vertically distributed database into clusters, determine when a new fragmentation is needed, and partition the database accordingly. The works presented show different experiments that were carried out to study the performance of both, using the TPC-H benchmark in a cluster of computers. The results of the experiment showed that SMOPD-C and SMOPD are able to perform dynamic fragmentation with high precision and to obtain a lower cost in the execution of queries compared to other approaches.

Alagiannis, Idreos & Ailamaki in [29] presented the H_2_O system that allowed the flexibility to support multiple storage designs and data access patterns on a single-engine. Plus, it decides on-the-fly, i.e., during query processing, it chooses the best design for a specific workload. A detailed H_2_O analysis was presented using the SDSS (Sloan Digital Sky Survey) benchmark. It was shown that while existing systems cannot achieve maximum performance across all workloads, H_2_O can always match best-case performance without requiring any tuning knowledge or specific workload.

The authors in [33] expanded the work in GridFormation, assigning the partition task to an RL (Reinforcement Learning) task. The proposal was experimentally validated using a database and a workload with the TPC-H benchmark and the Google Dopamine framework for deep RL. Competitive execution times were presented while increasing the number of attributes in a table, outperforming some cutting-edge algorithms.

Sharify et al. in [35] addressed the different challenges present in unstructured data through a lightweight relational database engine prototype and a flexible vertical partition algorithm that used simple heuristics to tailor the data design to the workload. Experimental evaluation using the Nobench dataset for JSON (JavaScript Object Notation) data showed that Argo and Hyrise, next-generation vertical partition algorithms, were outperformed by 24%. Furthermore, the proposed algorithm was able to achieve around 40% better cache utilization and 35% better Translation Lookaside Buffer (TLB) utilization.

Schroeder et al. [37] presented an RDF data distribution method that overcomes the shortcomings of current approaches to scale RDF storage in both data volume and query processing. This approach was implemented in a summary view of data to avoid exhaustive analysis of large data sets. As a result, the fragmentation templates were derived from data elements in an RDF structure. Additionally, an approach was provided for inserting dynamic data, even if the new data does not conform to the original RDF structure.

#### 5.2.3. Dynamic Hybrid Fragmentation

Wang et al. [69] addressed the problem of data distribution using a general triangle model called DaWN (Data, Workload, and Nodes). Based on data and workload analysis, it presents a novel strategy called ADDS (Automatic Data Distribution Strategy) for automatic data distribution in OLTP applications. The evaluation of this approach was carried out using the TPC-C benchmark and the MySQL database. The authors compared three different strategies: Hashing, Round-Robin, and ADDS. Based on the results of a series of experiments on TPC-C data sets and transactions, the proposed approach shows effective improvements.

In [70], the authors proposed SOAP (System Framework for Scheduling Online Database Repartitioning), a framework of a system for scheduling refragmentation of online databases for OLTP workloads. SOAP serves the goal of minimizing the run time for refragmentation operations while ensuring the correctness and performance of the concurrent processing of normal transactions. PostgreSQL was prototyped and a comprehensive pilot study was conducted on Amazon EC2 (Elastic Compute Cloud) to validate the significant performance benefits of SOAP.

Kulba & Somov [71] analyzed the dynamic fragment allocation in distributed data processing systems. A heuristic algorithm was presented to place fragments based on the parameters of each system over time. Two main methods of data fragmentation are used: horizontal and vertical fragmentation. The authors presented a method for the dynamic redistribution of table fragments between the nodes of a distributed system, taking into account the current values of the system parameters, which can change over time.

#### 5.2.4. Other Dynamic Fragmentations

Jindal & Dittrich [68] presented AutoStore: an autotuning data store that monitors current workload and automatically splits data into control time intervals, without human intervention. This allowed AutoStore to adapt to workloads without stopping the system. The experimental results were obtained using the TPC-H benchmark data set and showed that AutoStore outperformed the row and column designs by up to a factor of 2.

Sleit et al. [80] improved the ADRW (Adaptive Distributed Request Window) algorithm to achieve dynamic fragmentation and object allocation in distributed databases. The main result of the experiments carried out is that E-ADRW (Enhance ADRW) required less storage space compared to two other algorithms shown in the analysis stage.

Hung & Huang [81] proposed a new Dynamic Fragment Allocation Algorithm in Partially Replicated Allocation Scenario (DFAPR). To evaluate the algorithm, the OptorSim simulator was used and different sites were considered in MMDB (Main Memory Database). OptorSim performs 100 to 5000 operations with 6 types of work. The results shown in the simulation demonstrate that DFAPR is suitable for the MMDB cluster because it provides better response time and maximizes local processing.

Chernishev [91] presented an in-depth analysis of the prospects for an adaptive distributed relational column store. The column storage approach was shown to be a breakthrough in building an efficient self-managed database. As a conclusion, it was mentioned that different physical design options were presented to create the desired approach, as well as three alternatives to create an alert and execute the reorganization of the database.

### 5.3. Fragmentation for NoSQL DBMS

The authors in [57] used data mining and cluster analysis on the database records to apply data fragmentation. They compared the average response times of three related algorithms in a simple web application using a cloud-based NoSQL database management system. The experimental study shows that the presented techniques improve the performance of web applications.

Elghamrawy in [59,63] proposed an Adaptive Rendezvous Hashing Partitioning Module (ARHPM) for Cassandra NoSQL databases. To evaluate the proposed module, Cassandra was modified incorporating the partitioning module and a series of experiments was carried out to validate the load balancing of the proposed module using the Yahoo Cloud Serving Benchmark. The two experiments showed that the proposed algorithm is faster to fragment and a better scheme is obtained in terms of performance.

Oonhawat & Nupairoj [64] developed a new data distribution algorithm based on fragmentation conscious tagging to minimize the effect of the access point problem, especially in systems with heavy write requirements. As a conclusion, it was mentioned that the system improved because it is less likely that a certain fragment is affected during the consultations by the access point problem.

Heni & Gargouri [85] presented a new methodological approach to big data security based on data fragmentation. The proposed development shows four phases. The first phase is used to automatically group Big Data in the NoSQL database. The second phase consists of identifying sensitive data using a neural network. The third phase consists of providing a layer of security through fragmentation. The last layer is intended to rebuild the fragments. The tests were carried out on the MongoDB database, however, it is not detailed which data set was used.

In [92], the problem of using SSD (Solid-State Drive) flash drives was addressed. They introduced a new flow mapping scheme based on unique MongoDB features. The results that were obtained when evaluating the approaches, showed improvements in the performance of two benchmarks, YCSB (Yahoo! Cloud Serving Benchmark) and Linkbench.

Santos, Ghita & Masala [96] introduced an approach to data security in the cloud using a random pattern fragmentation algorithm and combining it with a distributed NoSQL database. The experiments were performed on a data set with four types of data. Each file was 100 kb in size and was stored on Cassandra. Cassandra was deployed on the Microsoft Azure infrastructure. The results showed a higher performance compared to their counterparts, which implies the usability of the proposed method in cloud computing, especially in scenarios with high-speed needs and limited resources.

### 5.4. Other Types of Fragmentation

This section groups together all the works that are not included in the other classifications, i.e., that do not focus on multimedia databases, that do not perform dynamic fragmentation, and that are not developed for NoSQL databases.

#### 5.4.1. Other Types of Horizontal Fragmentation

Castro et al. [3] showed an analysis of different methods of fragmentation, allocation, and replication of databases and a web application called FRAGMENT that adopted the design technique that was selected in the analysis stage, because the approach presented a method of fragmentation and replication, it was applied to a cloud environment, it was easy to implement, it focused on improving the performance of the operations executed in the database, it showed everything necessary for its implementation and it was based on a cost model. The experiments with the TPC-E benchmark demonstrated a lower response time of the queries executed against the distributed database generated by FRAGMENT compared to a centralized database.

The authors in [42] addressed the fragmentation and horizontally derived allocation simultaneously in the context of the complex data model. The results demonstrated that the presented heuristic approach for derived horizontal fragmentation improved system performance over other traditional fragmentation approaches.

Bellatreche et al. [44] developed a combined algorithm that handles the dependency problem between fragmentation and allocation. A new genetic solution was developed to solve this hurdle. Experiments for the genetic solution and previous work were carried out using the SSB benchmark (Star Schema Benchmark) applying it in Teradata with TD 13.10 software. The results showed that the genetic solution is faster than the previous work by 38%. A detailed description of the implementation of the proposed approach was presented, specifying that 22 queries were used, of which 50 selected predicates were obtained.

Lim [50] investigated the support for elastic data fragmentation in cloud-based parallel SQL processing systems. The author proposed different algorithms and associated data organization techniques that minimized the distribution of tuples and the movement of data between nodes. The experimental evaluation demonstrated the effectiveness of the proposed methods.

Islam khan [62] presented a technique called MMF (Matrix Based Fragmentation), which can be applied both in the initial stage and in the later stages of the design of distributed databases. To evaluate the approach, fragmentation was applied in a proposed scheme related to customer management. Through experiments, it was shown that the proposed technique achieved a success rate. For this reason, the performance of a distributed database management system is significantly improved by avoiding frequent remote access and high data transfer between sites.

#### 5.4.2. Other Types of Vertical Fragmentation

Fung, Karlapalem, and Li [23] the development of a comprehensive and analytical cost model for query processing in vertically fragmented object-oriented database classes. A set of results from analytical evaluations was presented to show the effect of vertical fragmentation and to study the relationship between the projection radius, the vis-a-vis sequential selectivity factor, and access by index. Subsequently, the implementation of an experimental prototype that allows the vertical fragmentation of classes in a commercial object-oriented database to validate the cost model was shown. A structure of classes related to employees was fragmented, the classes are “Emp”, “Dept” and “Proj”, with 1000, 200, and 1000 instances respectively.

The authors of [23] developed in [25] a heuristic algorithm called HCHA (Hill-Climbing Heuristic Algorithm) that takes the solution given by an affinity-based algorithm and improves it, thus reducing the total number of disk accesses. Furthermore, a second cost-based algorithm was developed and HCHA was shown to be significantly more efficient than the cost-based approach. The experiments were carried out in an object-oriented database containing employee-related data.

A new algorithm called CHAC (Column-oriented Hadoop based Attribute Clustering) was proposed in [27] to design an appropriate attribute grouping algorithm to achieve optimal data processing performance in the column-oriented Hadoop environment. To perform the tests, the TPC-H benchmark was used and the algorithm was evaluated in 16 nodes of which one of them acted as the master. The database contained 30 attributes and 20 GB in size. It was observed that the results generated by the cost model are closely related to the execution time of the queries in the mapping phases since their trend is consistent, which indicates the effectiveness of the proposed cost model.

Zhao et al. [31] provided a linear mixed-integer programming optimization formulation that was shown to be NP-hard. A heuristic was designed with two stages that find a solution close to the optimal solution in a fraction of the time. Optimization formulation and heuristics were extended for linear raw data processing, a scenario in which access and data extraction are performed simultaneously. For the implementation, the SDSS benchmark was used to evaluate the system with real data and the 100 most popular queries from the photoPrimary table were selected.

Costa, Costa, and Santos [34] evaluated the impact of partitioning and data storage in Hive-based systems, testing different data organization strategies and verifying the efficiency of these strategies on query performance. As a conclusion, it was mentioned that the implementation of strategies based on fragmentation brings benefits both in terms of storage and in terms of query processing.

Amer [36] introduced a k-means heuristic approach to vertical fragmentation and allocation. This approach was primarily focused on the early stage of DDBS (Distributed Database Systems) design. A short but effective experimental study was carried out, both on artificially created and real data sets, to demonstrate the optimization of the proposed approach against its counterparts. The results obtained supported that the work shown by the author surpassed different works in the experimentation stage.

#### 5.4.3. Other Types of Hybrid Fragmentation

Al-Kateb et al. [72] presented the main features of the Teradata approach and explained in detail a new approach to implementing row-column storage. Subsequently, a performance study was presented that demonstrates how different fragmentation options affect query performance, and different query optimization techniques are proposed specifically applicable to fragmented tables. The deployment took place on the Teradata 6650 Enterprise Data Warehouse. The TPC-H benchmark was used with a terabyte in size.

Campero Durand et al. [75] considered the feasibility of a general machine learning solution to overcome the drawbacks of more common approaches to fragmentation. The work in GridFormation was extended, assigning the partition task to an RL (Reinforcement Learning) task. The proposal was validated experimentally using a database and a workload with the TPC-H benchmark and the Google Dopamine framework for deep RL. Competitive runtimes were featured while increasing the number of attributes in a table, outperforming some cutting edge algorithms.

Schreiner et al. [76] proposed an automated approach to hybrid data fragmentation that automatically reorganizes the data based on the current workload of the NewSQL databases. The authors concluded by mentioning that the proposed work is unprecedented in the literature as it is the only research that proposes a hybrid fragmentation approach, offering data storage and optimization based on access workload.

H2TAP (Heterogeneous Hybrid Transactional Analytical Processing) has been developed to match the requirements for low-latency analysis of real-time operational data. Pinnecke et al. [79] proposed different solutions to many of these challenges in isolation: a unified engine has not yet been developed to optimize performance when combining these solutions. The authors suggested a highly flexible and adaptable data structure called GRIDTABLE to physically organize sparse but structured records in the context of H2TAP. The experiments were carried out using the CUSTOMER and LINEITEM tables of the TPC-C benchmark.

#### 5.4.4. Other Types of Fragmentation

Cuzzocrea et al. [83] introduced the use of an algorithm based on K-means clustering for effective and efficient support of the fragmentation of large XML data stores, and at the same time, control and determination of the number of fragments originated through the configuration of the adequate value of the K parameter. To validate the approach, the fragmentation strategy was compared with two significant adaptations of the two most common fragmentation methods for storing relational data, the PC (Predicate Construction) and AB (Affinity-based) fragmentation techniques. The experimental results showed that the proposed approach surpasses both comparison techniques under a certain perspective of the experimental analysis.

Turcu et al. [90] determined optimal fragmentation schemes, which greatly aid the design of schemes when dealing with non-trivial amounts of data. The development was implemented in the DTM (Distributed Transactional Memory) system and the tests were carried out under the TPC-C, TPCW, AuctionMark, EPinions, and ReTwis benchmarks in a distributed system of 20 physical computers with the H-base database management system. To validate the development, 5 benchmarks were used and, in most cases, improvements were observed both in the proportion of distributed transactions and in transactional performance.

Khan et al. [93] presented a robust, fault-tolerant, and scalable cluster-wide deduplication that was able to eliminate duplicate copies across the entire cluster. The evaluation showed great savings in disk space with minimal performance degradation, as well as great robustness in the event of sudden server failure. The approach was implemented in Ceph, a cluster with shared-nothing architecture. The FIO (Flexile I/O Tester) benchmark was used with 500 GB of workload.

## 6. Discussion

In this work, extensive research was carried out in the state of the art of data fragmentation, obtaining the most related approaches, classifying them, and describing each one of them. It is observed that fragmentation methods proposed in [32,56,73] present simple cost models for their implementation. [32,56] carry out a dynamic vertical and horizontal fragmentation respectively. Rodríguez-Mazahua et al. [73] developed a hybrid fragmentation technique that stands out for containing a simple cost model focused on multimedia databases. The three mentioned works present, for vertical, horizontal, and hybrid fragmentation, excellent ways to carry out fragmentation and are considered in a special way in this work, since they stand out for various characteristics, i.e., easy implementation, they provide a cost model, completeness (authors include all the information required to reproduce their methods), they consider multimedia data to obtain the fragmentation scheme and they take into account dynamic fragmentation.

It was observed that the year of publication of various papers ([1,2,22,23,24,25,38,39,40,41,42,43,80,82,83]) was more than 10 years ago and some of these articles have not been cited in the last five years ([2,22,38,39,42,82,83]). Therefore, these approaches are considered out of date.

The authors address different types of fragmentation in each analyzed work. Figure 5 shows the number of proposals related to each type of fragmentation. Articles [1,2,3,38,39,40,41,42,43,44,45,46,47,48,49,50,51,52,53,54,55,56,57,58,59,60,61,62,63,64,65,66] focus on horizontal fragmentation; while [7,22,23,24,25,26,27,28,29,30,31,32,33,34,35,36,37] consider vertical partitioning, [67,68,69,70,71,72,73,74,75,76,77,78,79] perform hybrid fragmentation, and [80,81,82,83,84,85,86,87,88,89,90,91,92,93,94,95,96,97] take into account other types of fragmentation.

The most relevant work in the category “Fragmentation of Multimedia Databases” is [1] since it has been cited in several papers ([26,32,40,41,55,67,73]); in contrast, the most influential method in the category “Dynamic Fragmentation” is [70] because it has motivated a number of approaches ([28,29,30,31,32,35,52,56,60,66,69,70,75,77,79,91]); in the category “Fragmentation for NOSQL DBMS”, the article that has attracted more attention is [63]; finally, [25] is the most cited work in the category “Other Types of Fragmentation” ([23,26,27,32]).

## 7. Conclusions and Future Work

A large number of works are observed in which fragmentation is used to improve the performance of databases using different techniques and solving various aspects. In this article, the research works related to dynamic fragmentation in multimedia databases were reviewed and classified into four categories: (1) For multimedia databases; (2) For dynamic databases; (3) For NoSQL databases; and (4) Others. Some categories were sub-classified by the type of fragmentation that occurs in the works. It is concluded that dynamic fragmentation for multimedia databases is a topic of great interest in the area of databases since it achieves good results in performance applying it in different ways. However, current information trends point to a large amount of multimedia data and new ways to improve the response performance of such databases are required.

An in-depth analysis of all the works obtained was carried out and it was observed through these that the most used benchmark in the field of fragmentation was TPC-H; Oracle, MongoDB, and PostgreSQL were the database management systems utilized in more works; the most considered cost was the cost of transportation; horizontal fragmentation was the most applied technique; Springer was the editorial with the highest number of articles, and 2019 was the year in which the most works were found. The importance of this research is that it can provide researchers and practitioners with an overview of the state of the art in database fragmentation.

As future work, this research will lay the foundations for the development of a Web application based on the main features obtained throughout this work and focused on dynamic fragmentation and multimedia databases.

## Figures and Tables

**Table 1:** Description of articles obtained in the search stage.

Article	Type of Fragmentation	Cost Model	Dynamic	CBIR	Cost Equations
[22]	Vertical	Yes	Yes	No	$\begin{array}{c} \min z=\underset{k}{\sum }\underset{i}{\sum }f_{ki}\underset{m}{\sum }\underset{j}{\sum }q_{km}l_{km}C_{ij}x_{mj}\\ \;+\underset{i}{\sum }\underset{k}{\sum }\underset{j}{\sum }c_{1}f_{ki}y_{kj}\\ \;+\underset{j}{\sum }c_{2}w_{j}\\ \;+\underset{m}{\sum }\underset{i}{\sum }\underset{j}{\sum }a_{mi}c_{ij}d_{m}x_{mj} \end{array}$
[23]	Vertical	Yes	No	No	Total_cost = IO_cost + CPU_cost
[24]	Vertical	Yes	No	No	Total cost = IO cost + CPU cost
[25]	Vertical	Yes	No	No	Total cost = Disk IO cost + CPU cost
[26]	Vertical	Yes	No	No	cost(vps_i_) = IAAC(vps_i_) + TC(vps_i_)
[27]	Vertical	Yes	No	No	$Min\left(\underset{q\in Q}{\sum }f_{q}*T\left(q,C\right)\right)$
[28]	Vertical	Yes	Yes	No	Not shown
[7]	Vertical	Yes	Yes	No	Not shown
[29]	Vertical	Yes	Yes	No	$q\left(L\right)=\overset{\left|L\right|}{\underset{i=1}{\sum }}\max\left(cost_{i}^{IO},cost_{i}^{CPU}\right)$
[30]	Vertical	Yes	Yes	No	Not shown
[31]	Vertical	Yes	No	No	Not shown
[32]	Vertical	Yes	Yes	No	cost(vps_i_) = IAAC(vps_i_) + TC(vps_i_)
[33]	Vertical	Yes	No	No	Not shown
[34]	Vertical	No	No	No	Not shown
[35]	Vertical	Yes	Yes	No	$C^{P}=\alpha \times \frac{CPC^{p}}{CPC^{max}}+\left(1-\alpha \right)\times \frac{RAC^{p}}{RAC^{max}}$
[36]	Vertical	Yes	No	No	$\begin{array}{c} Similarity\left(Q_{k1},Q_{k2}\right)\\ =\overset{q}{\underset{k1=1}{\sum }}\overset{q}{\underset{k2=1}{\sum }}(1-difference\left(P\left(\left(Q_{k1}\right),P\left(Q_{k2}\right)\right)\right)) \end{array}$
[37]	Vertical	Yes	Yes	No	Not shown
[2]	Horizontal	No	Yes	No	Not shown
[38]	Horizontal	Yes	Yes	No	Not shown
[1]	Horizontal	Yes	No	Yes	Not shown
[39]	Horizontal	Yes	No	No	$\overset{m}{\underset{j=1}{\sum }}cost_{\lambda }\left(Q_{j}\right)\cdot frec_{j}$
[40]	Horizontal	No	No	Yes	Not shown
[41]	Horizontal	No	No	Yes	Not shown
[42]	Horizontal	Yes	No	No	$\overset{m}{\underset{j=1}{\sum }}cost_{\lambda }\left(Q_{j}\right)\cdot frec_{j}$
[43]	Horizontal	Yes	Yes	No	utilityMigrate = card(SW(S_r_)) − card(SW(S_l_)) − card(F)
[44]	Horizontal	Yes	No	No	Not shown
[45]	Horizontal	Yes	Yes	No	Minimize TC = RC+ UC + SC
[46]	Horizontal	No	Yes	No	Not shown
[47]	Horizontal	Yes	Yes	No	$Cost\left(Q,SF\right)=\overset{t}{\underset{k=1}{\sum }}Cost\left(Q_{k},SF\right)$
[48]	Horizontal	Yes	Yes	No	$\begin{array}{c} IOC\left(Q_{k},f_{i}\right)=\overset{N_{i}}{\underset{j=1}{\sum }}V\left(Q_{k},f_{i}\right)\overset{M_{j}}{\underset{i=1}{\prod }}Sel_{F}^{p_{j}}\times \lceil \frac{\left|\left|F\right|\right|\times L}{PS}\rceil \end{array}$
[49]	Horizontal	No	No	Yes	Not shown
[50]	Horizontal	Yes	No	No	Not shown
[51]	Horizontal	No	Yes	No	Not shown
[52]	Horizontal	Yes	Yes	No	Not shown
[53]	Horizontal	No	Yes	No	Not shown
[54]	Horizontal	Yes	Yes	No	$C_{ij}=\overset{p}{\underset{k=1}{\sum }}\overset{n}{\underset{\begin{array}{c} j^{\prime }=1\\ j^{\prime }\neq j \end{array}}{\sum }}cu_{kij^{\prime }}+cm_{ij}+\overset{p}{\underset{k=1}{\sum }}cal_{kij}$
[55]	Horizontal	Yes	No	No	cost(hps_i_) = ITAC(hps_i_) + TC(hps_i_)
[56]	Horizontal	Yes	Yes	No	$pCost\left(nSc,\;oSc\right)=\underset{o\in OtM}{\sum }mCost\left(o\right)$
[57]	Horizontal	No	No	No	Not shown
[58]	Horizontal	Yes	Yes	No	CC(S_i_, S_j_) = cost of creating the data packet + cost of transmitting the data packet from site S_i_ to site S_j_
[59]	Horizontal	No	No	No	Not shown
[60]	Horizontal	Yes	Yes	No	$L_{P}\left(p\right)=\underset{\begin{array}{c} v\in V\\ P\left(v\right)=p \end{array}}{\sum }w\left(v\right)+\underset{\begin{array}{c} v\in V\\ u\in V\\ P\left(v\right)=p\\ \langle v,u\rangle \in E\\ P\left(u\right)\neq p \end{array}}{\sum }w\left(\langle v,u\rangle \right)\cdot k$
[61]	Horizontal	No	No	No	Not shown
[62]	Horizontal	Yes	No	No	Not shown
[63]	Horizontal	Yes	No	No	Not shown
[64]	Horizontal	No	No	No	Not shown
[65]	Horizontal	Yes	Yes	No	Not shown
[66]	Horizontal	Yes	Yes	No	$\begin{array}{c} E=\overset{T}{\underset{i=1}{\sum }}(\overset{i-1}{\underset{j=1}{\sum }}p_{j}\cdot C_{use,idx}+\left(1-\overset{i-1}{\underset{j=1}{\sum }}p_{j}\right)\\ \cdot (p_{i}\cdot C_{build,idx}+\left(1-p_{i}\right)C_{use,fs})) \end{array}$
[3]	Horizontal	Yes	No	No	Not shown
[67]	Hybrid	No	No	Yes	Not shown
[68]	Hybrid	Yes	Yes	No	Not shown
[69]	Hybrid	Yes	Yes	No	Not shown
[70]	Hybrid	Yes	Yes	No	$Cost=\sum_{\forall T_{i}}f_{i}\left(C_{i}\left(o\right)-C_{i}\left(p\right)\right)$
[71]	Hybrid	Yes	Yes	No	SOC = TSTF + CTR + RPC
[72]	Hybrid	Yes	No	No	Not shown
[73]	Hybrid	Yes	No	No	cost(hps_i_) = IDAC(hps_i_) + TC(hps_i_)
[74]	Hybrid	No	No	Yes	Not shown
[75]	Hybrid	Yes	No	No	Not shown
[76]	Hybrid	No	No	No	Not shown
[77]	Hybrid	Yes	Yes	Yes	Not shown
[78]	Hybrid	No	No	No	Not shown
[79]	Hybrid	Yes	No	No	Not shown
[80]	Of objects	Yes	Yes	No	$\begin{array}{c} Cost_{E-ADRW}\left(W_{o}^{pi}\right)\\ =\{\begin{array}{c} 1p_{i}\in A_{o}fragmented\\ \left|A_{o}\right|C_{d}+\left|A_{o}\right|\;p_{i}\notin A_{o}\;and\\ not\;replicated\;or\;\\ fragmented\\ \left(\left|A_{o}\right|-1\right)C_{d}+\left|A_{o}\right|\;p_{i}\in A_{o}\\ and\;replicated \end{array} \end{array}$
[81]	Of objects	Yes	Yes	No	$t=\sum t_{l}+\sum t_{r}$
[82]	Of documents	No	No	Yes	Not shown
[83]	Of documents	Yes	No	No	Not shown
[84]	Of documents	No	No	Yes	Not shown
[85]	Of documents	No	No	No	Not shown
[86]	Of documents	No	No	No	Not shown
[87]	Of documents	No	No	No	Not shown
[88]	Of video file	No	No	Yes	Not shown
[89]	Of Unstructured data	No	No	No	Not shown
[90]	K-way graph partitioning	Yes	No	No	Not shown
[91]	Of columns	No	Yes	No	Not shown
[92]	Internal	No	No	No	Not shown
[93]	Of metadata	No	No	Yes	Not shown
[94]	Of high dimensional vectors	No	Yes	Yes	Not shown
[95]	Grid fragmentation	No	No	No	Not shown
[96]	Random file fragmentation	No	No	No	Not shown
[97]	Of files	No	No	No	Not shown

**Table 2:** Types of Cost Included in the Papers Obtained.

Cost	Articles
Cost of access to irrelevant data	[26,55,73,83]
Query Processing Cost	[39,69,71,81,82]
Storage cost	[31,39,42,45,54,65,67,71,75,81,86,96]
Transport cost	[3,26,27,29,38,39,42,44,48,54,55,62,65,72,73,76,79,80,81,95]
Access cost	[22,23,24,25,26,29,31,32,35,36,38,43,48,60,66,79,81]
Execution cost	[23,24,25,29,30,32,33,35,37,41,47,48,52,56,68,70,73]
Update cost	[41,58,67,75,79,80]
Fragment creation computational cost	[1,50,56,68]
Network cost	[44]
Computational cost	[41,72]
Communication cost	[33,36,37,43,45,54,58,69,70,71,81,96]
Maintenance cost	[47]
Replication cost	[90]
Movement cost	[90]
Response time cost	[27,30]
Local access cost	[54,81]
Attribute usage cost	[28]
Operation type cost	[28]
Load cost	[66]
Migration cost	[60]
Distribution cost	[58]
Allocation cost	[77]
Overhead cost	[77]

**Table 3:** Most Used Benchmarks.

Benchmark	Articles
XWeb	[83]
OO7	[39,42]
THUMOS	[88]
TRECVID	[88]
CRM	[95]
TPC-E	[3]
TPC-H	[7,27,28,30,33,34,50,52,68,72,95]
SSB	[34,44,68]
Linkbench	[92]
YCSB	[59,63,76,92]
TPC-W	[76,90]
SPECWeb2009	[57]
TPC-C	[56,60,69,78,79,90]
AuctionMark	[90]
EPinions	[90]
Re-Twis	[90]
APB-1	[47,48]
Sloan Digital Sky	[29,31,46,51]
FIO	[93]
Fashion-MNIST	[94]
SIFT	[94]
GloVe	[94]
NoBench	[35]
OLTP-Bench	[78]
BSBM	[37]

## References

[1] Saad S., Tekli J., Chbeir R., Yetongnon K., Manolopoulos Y., Pokorny J., Sellis T.K. (2006). Towards Multimedia Fragmentation. Advances in Databases and Information Systems.

[2] Vazquez J. A dynamic virtual fragmentation method for query recovery optimization. Proceedings of the 20th International Conference of the Chilean Computer Science Society.

[3] Castro-Medina F., Rodriguez-Mazahua L., Lopez-Chau A., Abud-Figueroa M., Alor-Hernandez G. (2020). FRAGMENT: A Web Application for Database Fragmentation, Allocation and Replication over a Cloud Environment. IEEE Lat. Am. Trans..

[4] Abdalla H., Artoli A. (2019). Towards an Efficient Data Fragmentation, Allocation, and Clustering Approach in a Distributed Environment. Information.

[5] Tarun S., Batth R., Kaur S. A Review on Fragmentation, Allocation and Replication in Distributed Database Systems. Proceedings of the 2019 International Conference on Computational Intelligence and Knowledge Economy (ICCIKE).

[6] Rodríguez-Arauz M., Rodriguez-Mazahua L., Arrioja-Rodríguez M., Abud-Figueroa M., Peláez-Camarena S., Martínez-Méndez L. Design of a Multimedia Data Management System that Uses Horizontal Fragmentation to Optimize Content-based Queries. Proceedings of the Tenth International Conference on Advances in Information Mining and Management (IMMM 2020).

[7] Rodriguez L., Li X., Cuevas-Rasgado A., Garcia-Lamont F. DYVEP: An active database system with vertical partitioning functionality. Proceedings of the 10th IEEE International Conference on Networking, Sensing and Control (ICNSC 2013).

[8] Vu M., Lofstedt T., Nyholm T., Sznitman R. (2020). A Question-Centric Model for Visual Question Answering in Medical Imaging. IEEE Trans. Med. Imaging.

[9] Bir P., Balas V. A Review on Medical Image Analysis with Convolutional Neural Networks. Proceedings of the IEEE International Conference on Computing, Power and Communication Technologies (GUCON 2020).

[10] Tschandl P., Argenziano G., Razmara M., Yap J. (2018). Diagnostic accuracy of content-based dermatoscopic image retrieval with deep classification features. Br. J. Dermatol..

[11] Kucharczyk M., Hay G., Ghaffarian S., Hugenholtz C. (2020). Geographic Object-Based Image Analysis: A Primer and Future Directions. Remote Sens..

[12] Lorek D., Horbiński T. (2020). Interactive Web-Map of the European Freeway Junction A1/A4 Development with the Use of Archival Cartographic Sources. ISPRS Int. J. Geo-Inf..

[13] Cybulski P., Horbiński T. (2020). User Experience in Using Graphical User Interfaces of Web Maps. ISPRS Int. J. Geo-Inf..

[14] Oroud I. (2019). The annual surface temperature patterns across the Dead Sea as retrieved from thermal images. Arab. J. Geosci..

[15] Kavitha P.K., Vidhya Saraswathi P. (2020). Content based satellite image retrieval system using fuzzy clustering. J. Ambient Intell. Humaniz. Comput..

[16] Oroud I. (2019). The utility of thermal satellite images and land-based meteorology to estimate evaporation from large lakes. J. Great Lakes Res..

[17] Du A., Wang L., Cheng S., Ao N. (2020). A Privacy-Protected Image Retrieval Scheme for Fast and Secure Image Search. Symmetry.

[18] Li D., Bai X. Criminal Investigation Image Retrieval Based on Deep Learning. Proceedings of the International Conference on Computer Network, Electronic and Automation (ICCNEA 2020).

[19] Rashid A., Yassin A., Abdel Wahed A., Yassin A. (2020). Smart City Security: Face-Based Image Retrieval Model Using Gray Level Co-Occurrence Matrix. J. Inf. Commun. Technol..

[20] Lew M., Sebe N., Djeraba C., Jain R. (2006). Content-based multimedia information retrieval: State of the art and challenges. ACM Trans. Multimed. Comput. Commun. Appl..

[21] Traina A., Brinís S., Pedrosa G., Avalhais L., Traina C. (2019). Querying on large and complex databases by content: Challenges on variety and veracity regarding real applications. Inf. Syst..

[22] Pérez J., Pazos R., Frausto J., Romero D., Cruz L., Cairó O., Sucar L.E., Cantu F.J. (2000). Vertical Fragmentation and Allocation in Distributed Databases with Site Capacity Restrictions Using the Threshold Accepting Algorithm. MICAI 2000: Advances in Artificial Intelligence.

[23] Fung C., Karlapalem K., Li Q. (2002). An evaluation of vertical class partitioning for query processing in object-oriented databases. IEEE Trans. Knowl. Data Eng..

[24] Fung C., Leung E., Li Q. (2003). Efficient Query Execution Techniques in a 4DIS Video Database System for eLearning. Multimed. Tools Appl..

[25] Fung C., Karlapalem K., Li Q. (2003). Cost-driven vertical class partitioning for methods in object oriented databases. VLDB J. Int. J. Very Large Data Bases.

[26] Rodriguez L., Li X., Hameurlain A., Liddle S.W., Schewe K.D., Zhou X. (2011). A Vertical Partitioning Algorithm for Distributed Multimedia Databases. Database and Expert Systems Applications (DEXA 2011).

[27] Gu X., Yang X., Wang W., Jin Y., Meng D. CHAC: An Effective Attribute Clustering Algorithm for Large-Scale Data Processing. Proceedings of the 2012 IEEE Seventh International Conference on Networking, Architecture, and Storage.

[28] Li L., Gruenwald L. Self-managing online partitioner for databases (SMOPD) a vertical database partitioning system with a fully automatic online approach. Proceedings of the 17th International Database Engineering & Applications Symposium (IDEAS 2013).

[29] Alagiannis I., Idreos S., Ailamaki A. H_2_O: A hands-free adaptive store. Proceedings of the 2014 ACM SIGMOD International Conference on Management of Data (SIGMOD 2014).

[30] Li L., Gruenwald L. SMOPD-C: An autonomous vertical partitioning technique for distributed databases on cluster computers. Proceedings of the 2014 IEEE 15th International Conference on Information Reuse and Integration (IEEE IRI 2014).

[31] Zhao W., Cheng Y., Rusu F. Vertical partitioning for query processing over raw data. Proceedings of the 27th International Conference on Scientific and Statistical Database Management (SSDBM 2015).

[32] Rodríguez-Mazahua L., Alor-Hernández G., Li X., Cervantes J., López-Chau A. (2016). Active rule base development for dynamic vertical partitioning of multimedia databases. J. Intell. Inf. Syst..

[33] Campero Durand G., Piriyev R., Pinnecke M., Broneske D., Gurumurthy B., Saake G., Welzer T. (2019). Automated Vertical Partitioning with Deep Reinforcement Learning. ADBIS 2019: New Trends in Databases and Information Systems.

[34] Costa E., Costa C., Santos M. (2019). Evaluating partitioning and bucketing strategies for Hive-based Big Data Warehousing systems. J. Big Data.

[35] Sharify S., Lu A., Chen J., Bhattacharyya A., Hashemi A., Koudas N., Amza C. An Improved Dynamic Vertical Partitioning Technique for Semi-Structured Data. Proceedings of the 2019 IEEE International Symposium on Performance Analysis of Systems and Software (ISPASS 2019).

[36] Amer A. (2020). On K-means clustering-based approach for DDBSs design. J. Big Data.

[37] Schroeder R., Penteado R., Hara C. (2020). A data distribution model for RDF. Distrib. Parallel Databases.

[38] Pinto D., Torres G. (2002). On Dynamic Fragmentation of Distributed Databases Using Partial Replication. Advances in Systems Theory, Mathematical Methods and Applications.

[39] Ma H., Schewe K., Wang Q. A heuristic approach to cost-efficient fragmentation and allocation of complex value databases. Proceedings of the 17th Australasian Database Conference (ADC 2006).

[40] Getahun F., Tekli J., Atnafu S., Chbeir R. The use of semantic-based predicates implication to improve horizontal multimedia database fragmentation. Proceedings of the Workshop on Multimedia Information Retrieval on the Many Faces of Multimedia Semantics (MS 2007).

[41] Tekli J., Getahun F., Atnafu S., Chbeiru R. Towards efficient horizontal multimedia database fragmentation using semantic-based predicates implication. Proceedings of the XXII Simpósio Brasileiro de Banco de Dados.

[42] Ma H., Schewe K., Wang Q. A heuristic approach to cost-efficient derived horizontal fragmentation of complex value databases. Proceedings of the eighteenth conference on Australasian database (ADC 2007).

[43] Hauglid J., Ryeng N., Nørvåg K. (2010). DYFRAM: Dynamic fragmentation and replica management in distributed database systems. Distrib. Parallel Databases.

[44] Bellatreche L., Benkrid S., Ghazal A., Crolotte A., Cuzzocrea A., Xiang Y., Cuzzocrea A., Hobbs M., Zhou W. (2011). Verification of Partitioning and Allocation Techniques on Teradata DBMS. Algorithms and Architectures for Parallel Processing.

[45] Abdalla H., Amer A. Dynamic horizontal fragmentation, replication and allocation model in DDBSs. Proceedings of the 2012 International Conference on Information Technology and e-Services.

[46] Liroz-Gistau M., Akbarinia R., Pacitti E., Porto F., Valduriez P., Liddle S.W., Schewe K.D., Tjoa A.M., Zhou X. (2012). Dynamic Workload-Based Partitioning for Large-Scale Databases. Database and Expert Systems Applications.

[47] Bellatreche L., Bouchakri R., Cuzzocrea A., Maabout S., Hameurlain A., Rahayu W., Taniar D. (2013). Incremental Algorithms for Selecting Horizontal Schemas of Data Warehouses: The Dynamic Case. Data Management in Cloud, Grid and P2P Systems.

[48] Derrar H., Nacer M., Boussaid O. (2013). Exploiting data access for dynamic fragmentation in data warehouse. Int. J. Intell. Inf. Database Syst..

[49] Fasolin K., Fileto R., Krugery M., Kasterz D.S., Ferreirax M.R.P., Cordeirox R.L.F., Trainax A.J.M., Traina C. Efficient Execution of Conjunctive Complex Queries on Big Multimedia Databases. Proceedings of the 2013 IEEE International Symposium on Multimedia.

[50] Lim L. Elastic data partitioning for cloud-based SQL processing systems. Proceedings of the 2013 IEEE International Conference on Big Data.

[51] Liroz-Gistau M., Akbarinia R., Pacitti E., Porto F., Valduriez P., Hameurlain A., Küng J., Wagner R. (2013). Dynamic Workload-Based Partitioning Algorithms for Continuously Growing Databases. Transactions on Large-Scale Data- and Knowledge-Centered Systems XII.

[52] Herrmann K., Voigt H., Lehner W. Cinderella—Adaptive online partitioning of irregularly structured data. Proceedings of the 2014 IEEE 30th International Conference on Data Engineering Workshops.

[53] Kumar R., Gupta N. An extended approach to Non-Replicated dynamic fragment allocation in distributed database systems. Proceedings of the 2014 International Conference on Issues and Challenges in Intelligent Computing Techniques (ICICT 2014).

[54] Baron C., Iacob N. (2014). A New Dynamic Data Fragmentation and Replication Model in DDBMSs. Cost Functions. Knowl. Horiz..

[55] Rodríguez-Mazahua L., Alor-Hernández G., Abud-Figueroa M., Peláez-Camarena S.G., Gelbukh A., Espinoza F.C., Galicia-Haro S.N. (2014). Horizontal Partitioning of Multimedia Databases Using Hierarchical Agglomerative Clustering. Nature-Inspired Computation and Machine Learning.

[56] Fetai I., Murezzan D., Schuldt H. Workload-driven adaptive data partitioning and distribution—The Cumulus approach. Proceedings of the 2015 IEEE International Conference on Big Data (Big Data).

[57] Sauer B., Hao W. Horizontal cloud database partitioning with data mining techniques. Proceedings of the 12th Annual IEEE Consumer Communications and Networking Conference (CCNC 2015).

[58] Abdel Raouf A., Badr N., Tolba M., Hassanien A., Mostafa Fouad M., Manaf A., Zamani M., Ahmad R., Kacprzyk J. (2016). Distributed Database System (DSS) Design Over a Cloud Environment. Multimedia Forensics and Security.

[59] Elghamrawy S., Hassanien A., Shaalan K., Gaber T., Azar A., Tolba M. (2016). An Adaptive Load-Balanced Partitioning Module in Cassandra Using Rendezvous Hashing. Proceedings of the International Conference on Advances in Intelligent Systems and Informatics.

[60] Serafini M., Taft R., Elmore A., Pavlo A., Aboulnaga A., Stonebraker M. (2016). Clay: Fine-grained adaptive partitioning for general database schemas. Proc. VLDB Endow..

[61] Wu Q., Chen C., Jiang Y. Multi-source heterogeneous Hakka culture heritage data management based on MongoDB. Proceedings of the Fifth International Conference on Agro-Geoinformatics (Agro-Geoinformatics 2016).

[62] Khan S. (2016). Efficient Partitioning of Large Databases without Query Statistics. Database Syst. J..

[63] Elghamrawy S., Hassanien A. (2017). A partitioning framework for Cassandra NoSQL database using Rendezvous hashing. J. Supercomput..

[64] Oonhawat B., Nupairoj N. Hotspot management strategy for real-time log data in MongoDB. Proceedings of the 19th International Conference on Advanced Communication Technology (ICACT 2017).

[65] Zar Lwin N., Naing T. Non-Redundant Dynamic Fragment Allocation with Horizontal Partition in Distributed Database System. Proceedings of the International Conference on Intelligent Informatics and Biomedical Sciences (ICIIBMS 2018).

[66] Olma M., Karpathiotakis M., Alagiannis I., Athanassoulis M., Ailamaki A. (2019). Adaptive partitioning and indexing for in situ query processing. VLDB J..

[67] Chbeir R., Laurent D. (2010). Enhancing Multimedia Data Fragmentation. J. Multimed. Process. Technol..

[68] Jindal A., Dittrich J., Castellanos M., Dayal U., Lehner W. (2012). Relax and Let the Database Do the Partitioning Online. BIRTE 2011: Enabling Real-Time Business Intelligence.

[69] Wang X., Fan X., Chen J., Du X. (2014). Automatic Data Distribution in Large-scale OLTP Applications. Int. J. Database Theory Appl..

[70] Chen K., Cao Y., Zhou Y. Online Data Partitioning in Distributed Database Systems. Proceedings of the 18th International Conference on Extending Database Technology.

[71] Kulba V., Somov S. Dynamic Fragment Allocation in Distributed System with Time-Varying Parameters of its Operation. Proceedings of the 13th International Conference “Management of large-scale system development” (MLSD 2020).

[72] Al-Kateb M., Sinclair P., Au G., Ballinger C. (2016). Hybrid row-column partitioning in teradata^®^. Proc. VLDB Endow..

[73] Rodríguez-Mazahua L., Alor-Hernández G., Cervantes J., López-Chau A., Sánchez-Cervantes J. (2016). A hybrid partitioning method for multimedia databases. DYNA.

[74] Mourão A., Magalhães J. (2017). Balancing search space partitions by sparse coding for distributed redundant media indexing and retrieval. Int. J. Multimed. Inf. Retr..

[75] Campero Durand G., Pinnecke M., Piriyev R., Mohsen M., Broneske D., Saake G., Sekeran M.S., Rodriguez F. GridFormation Towards Self-Driven Online Data Partitioning using Reinforcement Learning. Proceedings of the First International Workshop on Exploiting Artificial Intelligence Techniques for Data Management (aiDM 2018).

[76] Schreiner G., Duarte D., Santos Mello R. An Autonomous Hybrid Data Partition for NewSQL DBs. Proceedings of the 33rd Brazilian Symposium on Databases Companion.

[77] Vogt M., Stiemer A., Schuldt H. Polypheny-DB: Towards a Distributed and Self-Adaptive Polystore. Proceedings of the IEEE International Conference on Big Data (Big Data 2018).

[78] Schreiner G., Duarte D., Dal Bianco G., Mello R. A Hybrid Partitioning Strategy for NewSQL Databases. Proceedings of the 21st International Conference on Information Integration and Web-based Applications & Services (iiWAS 2019).

[79] Pinnecke M., Campero Durand G., Broneske D., Zoun R., Saake G. (2020). GridTables: A One-Size-Fits-Most H2TAP Data Store. Datenbank-Spektrum.

[80] Sleit A., AlMobaidee W., Al-Areqi S., Yahya A. (2007). A Dynamic Object Fragmentation and Replication Algorithm in Distributed Database Systems. Am. J. Appl. Sci..

[81] Hung T., Huang C. (2012). A Dynamic Data Fragmentation and Distribution Strategy for Main-Memory Database Cluster. Adv. Mater. Res..

[82] Torjmen M., Pinel-Sauvagnat K., Boughanem M. Towards a structure-based multimedia retrieval model. Proceedings of the 1st ACM international conference on Multimedia information retrieval (MIR 2008).

[83] Cuzzocrea A., Darmont J., Mahboubi H. (2009). Fragmenting very large XML data warehouses via K-means clustering algorithm. Int. J. Bus. Intell. Data Min..

[84] Torjmen-Khemakhem M., Pinel-Sauvagnat K., Boughanem M. (2013). Investigating the document structure as a source of evidence for multimedia fragment retrieval. Inf. Process. Manag..

[85] Heni H., Gargouri F., Arik S., Huang T., Lai W., Liu Q. (2015). A Methodological Approach for Big Data Security: Application for NoSQL Data Stores. Neural Information Processing.

[86] Santos N., Masala G., De Prieto G., Gallo L., Howlett R., Jain L., Vlacic L. (2018). Big Data Security on Cloud Servers Using Data Fragmentation Technique and NoSQL Database. Intelligent Interactive Multimedia Systems and Services.

[87] Mourão A., Magalhães J. Towards Cloud Distributed Image Indexing by Sparse Hashing. Proceedings of the International Conference on Multimedia Retrieval (ICMR 2019).

[88] Mettes P., van Gemert J., Cappallo S., Mensink T., Snoek C. Bag-of-Fragments: Selecting and encoding video fragments for event detection and recounting. Proceedings of the 5th ACM on International Conference on Multimedia Retrieval (ICMR 2015).

[89] Wycislik L., Gruca A., Brachman A., Kozielski S., Czachórski T. (2015). Independent Data Partitioning in Oracle Databases for LOB Structures. Man-Machine Interactions.

[90] Turcu A., Palmieri R., Ravindran B., Hirve S. (2016). Automated Data Partitioning for Highly Scalable and Strongly Consistent Transactions. IEEE Trans. Parallel Distrib. Syst..

[91] Chernishev G. (2017). The design of an adaptive column-store system. J. Big Data.

[92] Nguyen T., Lee S., Lee W., Choi W., Jung S., Song M. (2017). Optimizing MongoDB Using Multi-streamed SSD. Proceedings of the 7th International Conference on Emerging Databases.

[93] Khan A., Lee C., Hamandawana P., Park S., Kim Y. A Robust Fault-Tolerant and Scalable Cluster-Wide Deduplication for Shared-Nothing Storage Systems. Proceedings of the IEEE 26th International Symposium on Modeling, Analysis, and Simulation of Computer and Telecommunication Systems (MASCOTS 2018).

[94] Lu Y., Bo Y., He W., Nabatchian A. Dynamic Partition Forest: An Efficient and Distributed Indexing Scheme for Similarity Search based on Hashing. Proceedings of the IEEE International Conference on Big Data (Big Data 2018).

[95] Le T., Kantere V., Orazio L. Optimizing DICOM data management with NSGA-G. Proceedings of the International Workshop on Design, Optimization, Languages and Analytical Processing of Big Data.

[96] Santos N., Ghita B., Masala G. Enhancing Data Security in Cloud using Random Pattern Fragmentation and a Distributed NoSQL Database. Proceedings of the IEEE International Conference on Systems, Man and Cybernetics (SMC 2019).

[97] Sharma S., Bawa S. (2019). CBDR: An efficient storage repository for cultural big data. Digit. Scholarsh. Humanit..

[98] Son J., Kim M. (2004). An adaptable vertical partitioning method in distributed systems. J. Syst. Softw..

